# The Nuclear Effector of Wnt-Signaling, Tcf1, Functions as a T-Cell–Specific Tumor Suppressor for Development of Lymphomas

**DOI:** 10.1371/journal.pbio.1001430

**Published:** 2012-11-20

**Authors:** Machteld M. Tiemessen, Miranda R. M. Baert, Tom Schonewille, Martijn H. Brugman, Farbod Famili, Daniela C. F. Salvatori, Jules P. P. Meijerink, Ugur Ozbek, Hans Clevers, Jacques J. M. van Dongen, Frank J. T. Staal

**Affiliations:** 1 Department of Immunohematology and Blood Transfusion, Leiden University Medical Center, Leiden, The Netherlands; 2 Department Immunology, ErasmusMC, Rotterdam, The Netherlands; 3 Central Laboratory Animal Facility, Leiden University Medical Center, Leiden, The Netherlands; 4 Department of Pediatric Oncology/Hematology, Erasmus MC/Sophia's Children's Hospital, Rotterdam, The Netherlands; 5 Department of Genetics, Institute for Experimental Medicine, Istanbul University, Istanbul, Turkey; 6 Hubrecht Laboratory, Utrecht, The Netherlands; National Jewish Medical and Research Center/Howard Hughes Medical Institute, United States of America

## Abstract

The HMG-box factor Tcf1 is required during T-cell development in the thymus and mediates the nuclear response to Wnt signals. Tcf1^−/−^ mice have previously been characterized and show developmental blocks at the CD4−CD8− double negative (DN) to CD4+CD8+ double positive transition. Due to the blocks in T-cell development, Tcf1^−/−^ mice normally have a very small thymus. Unexpectedly, a large proportion of Tcf1^−/−^ mice spontaneously develop thymic lymphomas with 50% of mice developing a thymic lymphoma/leukemia at the age of 16 wk. These lymphomas are clonal, highly metastatic, and paradoxically show high Wnt signaling when crossed with Wnt reporter mice and have high expression of Wnt target genes *Lef1* and *Axin2*. In wild-type thymocytes, Tcf1 is higher expressed than Lef1, with a predominance of Wnt inhibitory isoforms. Loss of Tcf1 as repressor of Lef1 leads to high Wnt activity and is the initiating event in lymphoma development, which is exacerbated by activating Notch1 mutations. Thus, Notch1 and loss of Tcf1 functionally act as collaborating oncogenic events. Tcf1 deficiency predisposes to the development of thymic lymphomas by ectopic up-regulation of Lef1 due to lack of Tcf1 repressive isoforms and frequently by cooperating activating mutations in *Notch1*. Tcf1 therefore functions as a T-cell–specific tumor suppressor gene, besides its established role as a Wnt responsive transcription factor. Thus, Tcf1 acts as a molecular switch between proliferative and repressive signals during T-lymphocyte development in the thymus.

## Abstract 2

Tcf1 is known to function as a transcriptional activator of Wnt-induced proliferation during T cell development in the thymus. Evidence for an additional contrasting role for Tcf1 as a T-cell specific tumor suppressor gene is now presented.

## Author Summary

Cancers often develop as a consequence of deregulated expression of key factors that operate during normal development. T-cell factor 1 (Tcf1) has an established role in the nuclear response to Wnt signaling during normal T-cell development in the thymus. Here we show in mice that the absence of Tcf1 can trigger tumorigenesis. As expected from previous work, lack of Tcf1 results in a small thymus with several partial blocks in T-cell development in the thymus. Surprisingly, we observe that a large proportion of Tcf1^−/−^ mice spontaneously develop thymic lymphomas. Thorough investigation of these thymic-derived tumors revealed that the mechanism underlying these lymphomas is, paradoxically, increased levels of Wnt-signaling. We propose that Wnt-signaling in these tumors is mediated by up-regulated expression of the Tcf1-homologue, Lef1, and specifically its long isoform. Furthermore, we have evidence to propose that in a normal thymus, short isoforms of Tcf1 that cannot respond to Wnt signals act as repressors of Lef1-mediated Wnt-signaling. Thus, we propose that Tcf1 has a dual function developing T cells in mice: it functions as a T-cell–specific tumor suppressor gene in addition to its established role as a transcriptional activator of Wnt-induced proliferation. Whether loss of function of Tcf-1 as a tumor suppressor gene actually occurs in human T-cell lymphoblastic leukemias is currently under investigation.

## Introduction

Cancers often develop as consequence of deregulated expression of key factors that operate during normal development. Deregulation of the Wnt signaling pathway has been implicated in many types of malignancies, especially in solid tumors (reviewed in [1]–[3]). Mutations in different components of the Wnt pathway are found to contribute to carcinogenesis [3]. During normal development, Wnt proteins function as proliferation-inducing growth factors and may also affect cell-fate decisions [4]–[6]. Wnt proteins bind to their Frizzled receptors, thereby preventing proteosomal degradation of the Wnt mediator β-catenin. Subsequently, β-catenin is translocated to the nucleus, where it forms an active transcription complex with the nuclear proteins downstream of the Wnt pathway: TCF1 (T-cell factor 1, the product of the Tcf7 gene, referred to as Tcf1 throughout this article), LEF1 (Lymphocyte-Enhancer-binding Factor), or the homologous factors TCF4 and TCF3. All TCF/LEF factors belong to a family of high-mobility-group (HMG) proteins that utilize the HMG box for sequence-specific DNA binding. The HMG boxes of these factors are virtually identical and likely display indistinguishable DNA-binding specificities [7],[8]. TCF/LEF nuclear proteins exist as transcriptional repressors and only upon binding to β-catenin will form an active transcription complex. For TCF1, at least eight isoforms have been identified with different capacities to bind β-catenin, thereby influencing the responsiveness of cells toward Wnt signals [9]. The long isoforms of TCF1 contain the amino-terminal β-catenin-binding domain, whereas the shorter isoforms lack this domain and will therefore function as the naturally occurring repressors of the pathway.

A large body of evidence has shown that canonical Wnt signaling is essential for thymocyte proliferation and normal T-cell development [10]–[16]. Among the Wnt proteins, specifically Wnt1 and Wnt4 are essential for thymocyte proliferation [11], which is reflected in mice deficient for Wnt1 and Wnt4 that display low thymic cellularity [15]. In addition, overexpression of Wnt4 selectively expands thymic output from transduced hematopoietic stem cells [17]. Recently, we showed that another Wnt protein, Wnt3a, plays a crucial role in fetal thymopoiesis, with Wnt3a^−/−^ thymi showing severely reduced numbers of DP and a block of the preceding CD8+ Immature Single Positive (ISP) stage [18], thereby displaying an exact phenocopy of fetal thymi in Tcf1^−/−^ mice.

Studies on mice deficient for the Wnt-responsive nuclear proteins reveal crucial roles for Tcf1 in T-cell development and Lef1 in B-cell development [19],[20]. Tcf1^−/−^ mutant mice have a severe reduction of thymic cellularity and a partial block in thymocyte differentiation at the transition from the CD8+ ISP stage to the CD4+CD8+ double positive (DP) stage [19]. Thymocytes of Tcf1^−/−^ mice do not proliferate as strong as their wild-type counterparts [21]. These data indicate that lack of Tcf1 mainly results in lack of proliferation and therefore expansion of the thymocytes. Although Lef1^−/−^ mice have normal T-cell development, mice deficient in both Lef1 and Tcf1 have a complete block in T-cell differentiation at the ISP stage, which indicates redundancy between these two factors [22]. The block in T-cell development in Tcf1-deficient mice was shown to be caused by lack of Wnt mediated signals, as Tcf isoforms without the β-catenin binding domain could not restore T-cell development, but Tcf isoforms containing the interaction domain with the Wnt mediator β-catenin fully reconstituted T-cell development [23]. In addition, soluble Frizzled receptors acting as inhibitors of Wnt signaling [11], or overexpression of an inhibitor of the interaction between β-catenin and Tcf/Lef factors, ICAT [13], inhibited T-cell development at the same stages as Tcf1 KO mice. Recent studies by the laboratories of Bhandoola and Gounari further emphasize the importance of Tcf1 as a critical regulator of T-lineage specification and differentiation. These investigators demonstrate that Tcf1 is critical for induction of a T-cell-specific gene program in stem cells and uncommitted progenitors [16]. In addition, the Gounari lab showed that ETPs lacking Tcf1 fail to develop normally [10]. Together, these studies conclusively point to Tcf1 as an essential transcriptional regulator of T-cell specification, commitment, and lineage determination [24]. Here we report that Tcf1, besides acting as a Wnt responsive transcription factor, also has an important other function, namely as tumor suppressor for the development of T-cell lymphomas.

## Results

### Lack of Tcf1 Induces Thymic Lymphomas with High Frequency

**Figure 1:**
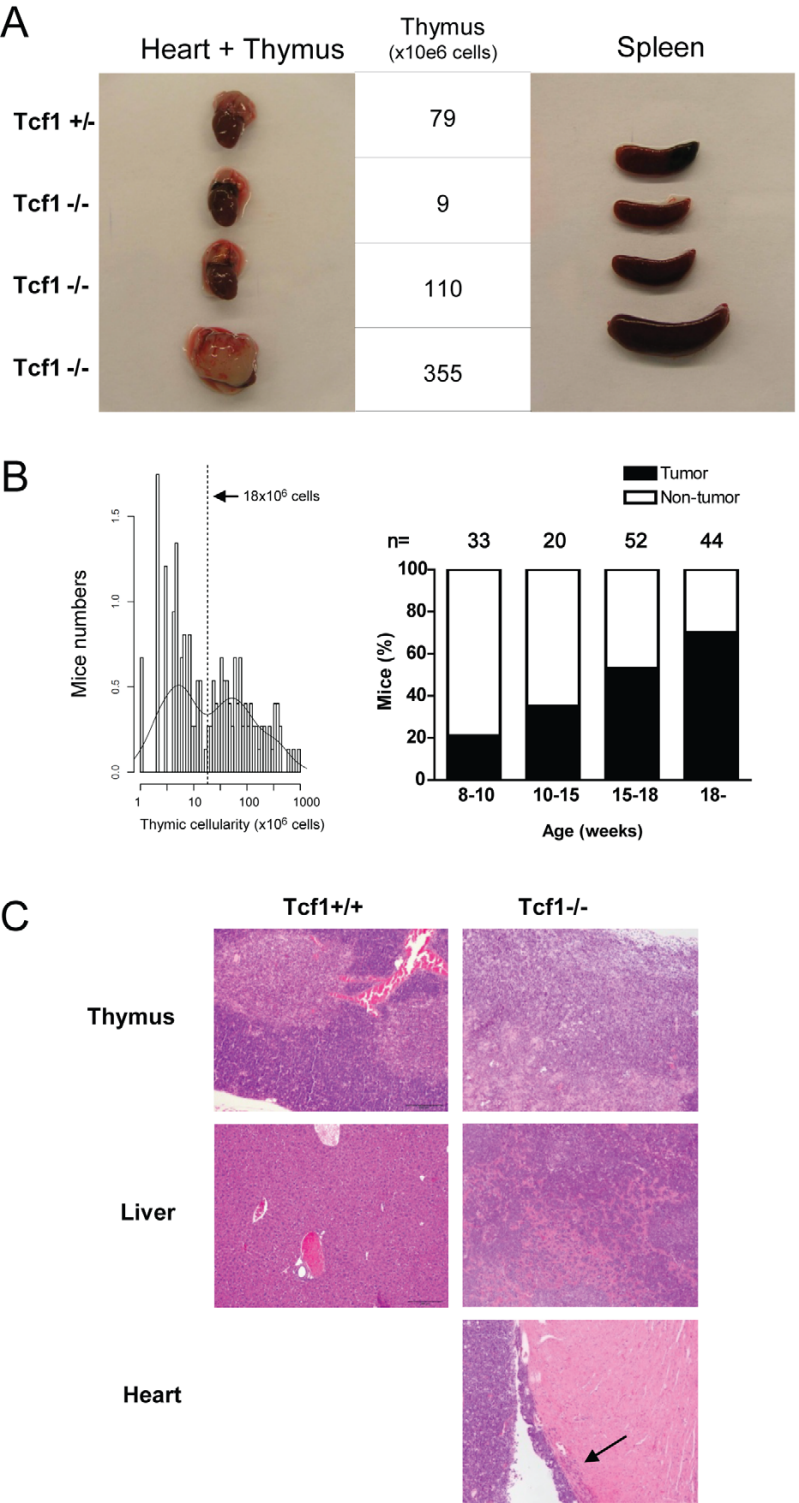
Tcf1^−/−^ mice develop thymic lymphomas. (A) Heart with thymus and spleen of one Tcf1^+/−^ and three Tcf1^−/−^ mice are shown. The number of thymocytes of the mice is from top to bottom: 79, 9, 110, and 355×10^6^ cells. (B) Tcf1^−/−^ mice are sacrificed at a certain age (8–10, 10–15, 15–18, or >18 wk), and according to the bimodal distribution of the thymus size, mice were considered to have a tumor when the thymus contains >18×10^6^ cells. In total 150 Tcf1^−/−^ mice were analyzed. The percentage of mice categorized to have a tumor is shown for each age group in the right panel. (C) Histopathology of normal tissue compared to tumors from Tcf1^−/−^ mice. Paraffin sections of thymus, liver (Tcf1^+/+^ and Tcf1^−/−^), and heart (Tcf1^−/−^ only) were stained with hematoxylin and eosin (HE). The HE sections of the Tcf1^−/−^ mouse show neoplastic cells arranged in cords and sheets in the thymus that infiltrate in the liver and the heart; final magnification, 100×.

**Figure 2:**
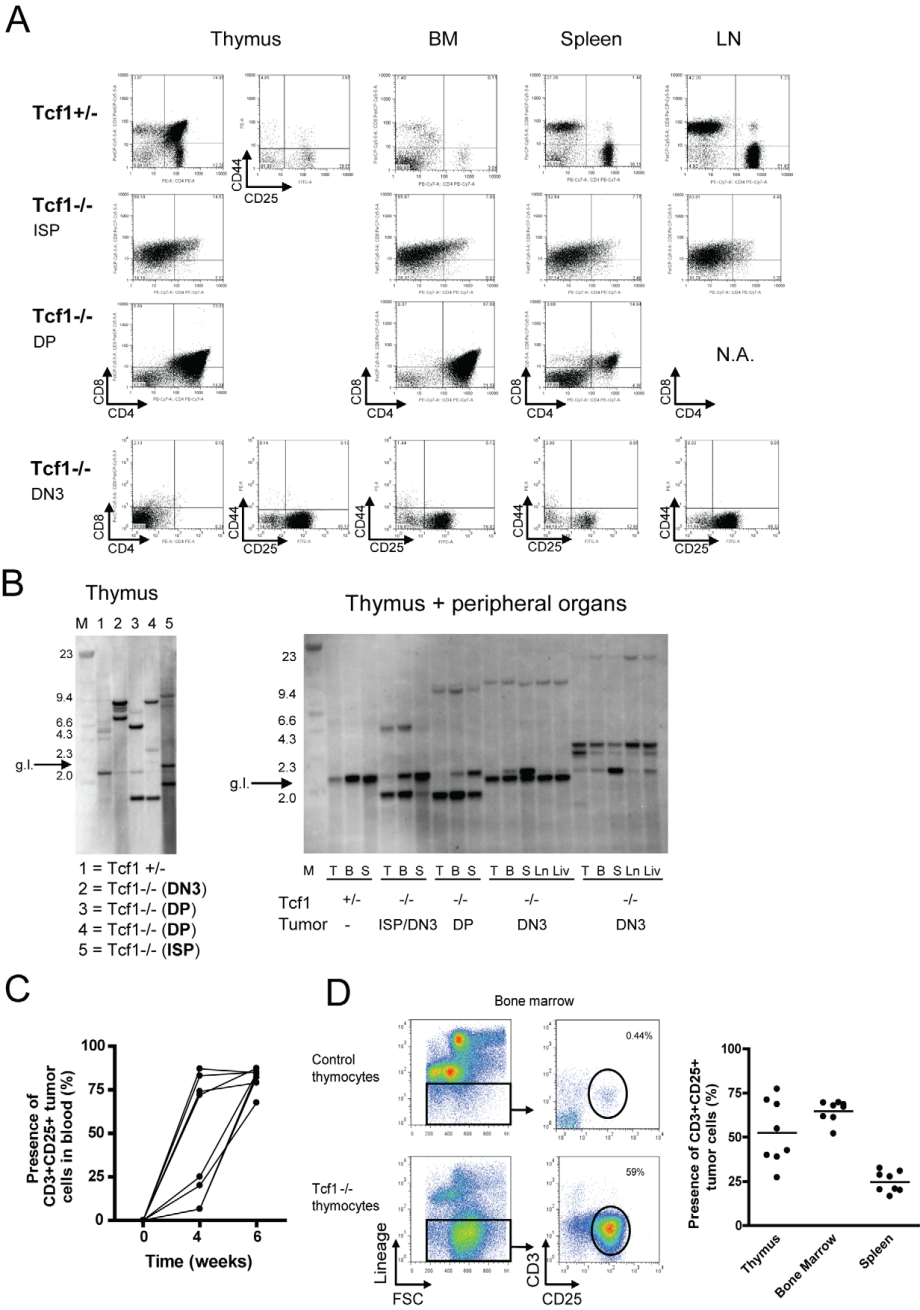
Tcf1^−/−^ thymic lymphomas are phenotypically heterogeneous, malignant, and oligoclonal. (A) Thymocytes, bone marrow (BM) cells, splenocytes, and lymphocytes obtained from lymph nodes (LN) were stained for CD4, CD8, CD44, and CD25. The CD4/CD8 and CD44/CD25 plot is shown for lineage negative cells for all the different organs. Results are shown for one Tcf1^+/−^ mouse and three representative Tcf1^−/−^ mice. (B) Left panel, genomic DNA was prepared from thymocytes derived from a Tcf1^+/−^ mouse (Lane 1, showing the germline band and a faint pattern of additional bands) and four different Tcf1^−/−^ mice with thymic lymphomas (DN3 tumor, Lane 2; DP tumors, Lanes 3 and 4) and an ISP tumor (Lane 5). The asterisk indicates the fragment expected for the germ-line (g.l.) TCRβ gene configuration. On the left side, a size marker (M) was included of which the sizes are indicated in the figure (kD). Right panel, genomic DNA was prepared from different organs, T (thymus), B (bone marrow), S (spleen), LN (lymph nodes), and Liv (liver) of one control Tcf1^+/−^ mouse and four different Tcf1^−/−^ mice. The phenotype of the lymphoma as determined by FACS analysis is shown for each mouse. The first lane includes a size marker (M) of which the sizes (kD) are indicated in the figure. (C) Tcf1^−/−^ tumor cells characterized by an intermediate expression of CD3 and CD25 were injected into sublethally irradiated Rag1^−/−^ mice by tail vein injection. Mice were bled at the time of injection, 4 and 6 wk after injection. The presence of CD3+CD25+ tumor cells in blood is indicated per mouse (*n* = 8). (D) Six weeks after transfer of the cells, the mice were sacrificed and the thymus, bone marrow, and spleen were analyzed for the presence of tumor cells. Cell suspensions were stained for lineage markers (Mac1, Gr1, B220, Ter119, and NK1.1) and CD4, CD8, CD3, CD44, and CD25. An example of the gating strategy is shown for a bone marrow sample. The percentage of CD3+CD25+ tumor cells is shown per mouse per organ.

The generation of mice lacking the Wnt-responsive factor Tcf1 revealed a crucial role for Tcf1 in T-cell development [19]. Tcf1^−/−^ mice have thymi characterized by low cellularity (fewer than 10^7^ cells at 6–8 wk of age, compared to >10^8^ cells in littermates [19]), which is due to the blocks at the DN and ISP developmental stages. Strikingly, over time an increasing number of Tcf1^−/−^ mice were found with an extremely enlarged thymus (example in Figure 1A). The occurrence of these enlarged thymi in Tcf1^−/−^ mice was not a rare finding. Studying the thymi of 150 Tcf1^−/−^ mice showed a clear bimodal distribution in thymic cellularity (Figure 1B). A threshold in thymocyte numbers occurs at 18×10^6^ thymocytes. A cellularity of <18×10^6^ cells can therefore be regarded as a normal size Tcf1^−/−^ thymus, whilst a thymus with a cellularity >18×10^6^ cells can be regarded as an abnormal enlarged thymus. The right graph of Figure 1B demonstrates an increasing percentage of Tcf1^−/−^ mice with a hyperplastic thymus with increasing age. This hyperproliferation could be caused by increased normal proliferation or by the presence of a clonal population of tumor cells. Results described below collectively demonstrate that these cells are neoplastic in nature and represent thymic lymphomas (Figures 1C and 2). Immunohistochemical analysis shows that neoplastic cells completely disrupted the thymic architecture (Figure 1C), and loss of corticomedullary demarcation was evident. Neoplastic cells invaded the thymic capsule, neighboring adipose tissue, thoracic organs, liver, kidney, spleen, and lymph nodes. Abdominal organs (liver and kidney) and lymphatic tissues (spleen and lymph nodes) have been shown to be preferential sites for metastasis of systemic lymphomas [25].

### Tcf1^−/−^ Induced Thymic Lymphomas Are Clonal and Highly Metastatic

The lymphomas have different phenotypic characteristics that to some extend reflect the developmental blocks. Thus, the different lymphomas in the Tcf1^−/−^ thymi were categorized into several phenotypically distinct subgroups or mixtures thereof: DN1, DN3, ISP, or the DP stage (examples of DN3, ISP, and DP lymphomas are shown in Figure 2A). The different phenotypes are not correlated to the age of the mice, and their frequency is: 5% DN1, 32.5% DN3, 40% ISP, and 22.5% DP (*n* = 40 Tcf1^−/−^ tumor mice). The thymocytes overpopulating the thymus were present in other hematopoietic organs such as spleen, bone marrow, and lymph nodes (Figure 2A), suggesting the high malignant capacity of these cells to invade other organs as expected from the size of the organs. To examine whether this aggressive proliferation of thymocytes was due to clonal expansion, a Southern Blot analysis was performed, using the Jβ2 region of the TCRβ gene. In contrast to DNA from a Tcf1^+/−^ control thymus, which shows the germline band (g.l. indicated by the arrow) and a heterogeneous mix of bands characteristic of a polyclonal cell population (Figure 2B, left panel, Lane 1), the lymphoma samples only showed between two and four distinct bands (germline and one or more rearranged alleles), indicating that they consisted of one or two independent clones (Figure 2B, left panel, Lanes 2–5). Interestingly, the same clonal band was found in the metastases in the secondary organs, BM, spleen, liver, and lymph nodes (Figure 2B, right panel). To further confirm the malignancy of these thymic lymphomas and their ability to grow autonomously and invade the organs in secondary recipients, 5×10^5^ thymocytes were transferred into sublethally irradiated Rag1^−/−^ recipients. The malignant donor thymocytes were derived from Tcf1^−/−^ mice, characterized by intermediate expression of CD3 and CD25, and control donor thymocytes were obtained from Tcf1^+/−^ mice. Four weeks after transfer, the tumor cells (as characterized by the expression of CD3 and CD25) were present in peripheral blood in 50% of the recipient mice (Figure 2C). Six weeks after transfer, all animals were sacrificed. Recipients receiving the malignant thymocytes of Tcf1^−/−^ origin all displayed an enlarged liver and spleen, and tumor cells were detectable by flow cytometry in all organs tested (thymus, BM, spleen; Figure 2D). Together these results demonstrate that a lack of Tcf1 predisposes mice to a high risk of developing thymic lymphomas, which are clonal and characterized by an aggressive metastatic phenotype. These results indicate that Tcf1 functions as a tumor suppressor gene in the thymus.

### Tcf1^−/−^ Thymic Lymphoma Cells Exhibit Deregulated Wnt Pathway

**Figure 3:**
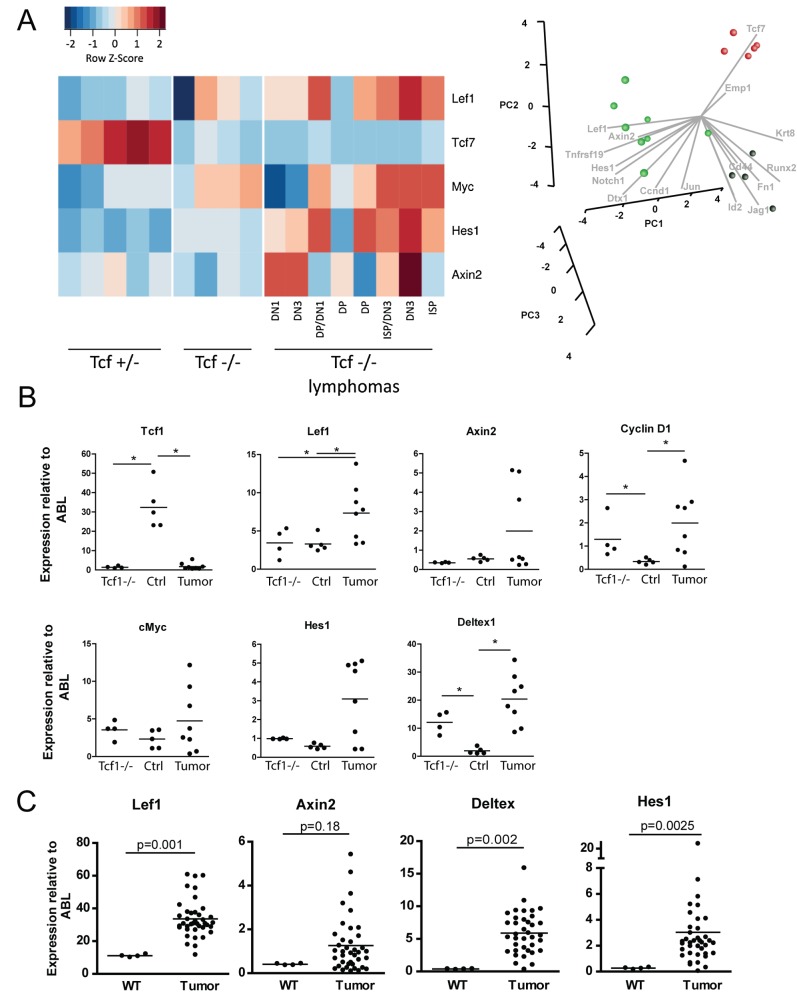
Tcf1^−/−^ lymphomas show deregulated Wnt signaling. (A) RNA isolated from thymi of 17 different mice was used for microarray analysis. Expression of Lef1, Tcf7, Myc, and Hes1 in the Tcf1^−/−^ mice without lymphoma (*n* = 4), Tcf1^+/−^ mice (control, *n* = 5) and Tcf1^−/−^ mice with a lymphoma (Lymphoma, *n* = 8) is shown. For the Tcf1^−/−^ lymphoma mice, the phenotype of the tumor is indicated on the horizontal axis. Columns represent independent RNA preparations of the different mice groups. A principal component analysis was performed using Wnt and Notch target genes. A PCA analysis shows clustering of the three groups as well as the effect each of the target genes has on the separation of these groups with samples of Tcf1^+/−^, Tcf1^−/−^, and Tcf1^−/−^ with tumors indicated by red, black, and green spheres, respectively. (B) Expression levels of Tcf1, Lef1, Axin2, Cyclin D1, cMyc, Hes1, and Deltex1 as determined by Affymetrix microarray were summarized and normalized using RMA, and the expression relative to Abl was plotted for each sample. Statistical significant differences (*p*<0.05) as determined by Mann–Whitney U test are indicated by an asterisk. (C) A panel of 40 Tcf1^−/−^ thymic lymphomas and four control thymi (Tcf1^+/−^) were analyzed by RQ-PCR. Expression data for Lef1 long (containing the β-catenin interacting domain), *Axin2*, *Deltex1*, and *Hes1* are shown relative to the house keeping gene *Abl*. Mann–Whitney U tests were performed to calculate the indicated *p* values.

To gain insight into the molecular mechanism underlying the Tcf1-deficient tumor development, we compared the gene expression profile of thymocytes derived from Tcf1^−/−^ mice with tumors, Tcf1^−/−^ mice of similar age without tumors, and control Tcf1^+/−^ mice. Samples of 17 mice were studied by genome-wide expression profiling using Affymetrix microarrays, namely five control Tcf1^+/−^ mice, four Tcf1^−/−^ mice without tumor, and eight thymic tumors from Tcf1^−/−^ mice. Expressions of several oncogenes, known to be involved in leukomogenesis, were analyzed and were not up-regulated in the thymic lymphomas compared to the Tcf1^−/−^ without lymphomas (*Tal1*, *Tal2*, *Lyl1*, *Lmo1*, *Lmo2*, *SilTal*, *p53*; unpublished data). Analysis of components of the Wnt pathway confirmed that *Tcf1* (*Tcf7*) expression was absent (as expected) in the Tcf1^−/−^ thymocytes (with and without a tumor), whilst in all but one Tcf1^−/−^ tumor sample, the expression level of the transcription factor Lef1 was up-regulated compared to control (Tcf1^+/−^) thymocytes (Figure 3A, left panel).

Principal component analysis of the Wnt target genes in all 17 thymic samples confirmed the obvious discriminating factor between Tcf1^−/−^ and Tcf1^+/−^ samples to be *Tcf7* (the HUGO gene name for Tcf1). The Tcf1^−/−^ tumor samples were clearly distinguished by factors involved in the Wnt-signaling pathway, *Axin2*, *Lef1*, and *Tnfrsf19*, or in the Notch signaling pathway, *Deltex1* and *Hes1* (Figure 3A, right panel). These results indicated that both Wnt and Notch signaling are affected in the Tcf1^−/−^ tumor samples compared to the other two groups. Tcf1^−/−^ samples without a tumor were distinguished by low expression of the following factors: *Emp1*, *Krt8*, *Runx2*, *CD44*, *Fn1*, *Jag1*, *Id2*, and *Cdh1*. Several of these genes are known to be Wnt target genes (*Runx2*, *Id2*, *CD44*, and *Fn1*). These data show ectopic up-regulation of Wnt signaling as demonstrated by high expression of *Lef1*, *CyclinD1*, and *c-Myc* as well as Notch target genes *Hes*1 and *Deltex1* (Figure 3B). Collectively, these data indicate that interaction between the Wnt and Notch pathways is necessary for full lymphomagenesis.

Confirmation of the array data was performed with a panel of 40 Tcf1^−/−^ thymic lymphomas by Q-PCR. In all tested tumor samples, the expression level of Lef1 was increased compared to thymocytes of control mice (Figure 3C). The mean expression Axin2 level of the 40 tumor samples was 4 times elevated compared to the mean expression Axin2 level of the control mice (1.2 versus 0.3), with 29 of the 40 tumor samples (73%) having a higher Axin2 level than 0.3 (Figure 3C). Moreover, the high *Axin2* expression in the majority (73%) of lymphomas in combination with the universally up-regulated Lef1 expression indicates a marked increase in Wnt signaling in these lymphomas. Further analysis of this panel of lymphomas showed that the expression levels of Hes1 and Deltex1, two target genes of Notch1 signaling, were enhanced in all tumor samples compared to the control samples (Figure 3C), again demonstrating that both the Wnt and Notch pathway are involved in full lymphomagenesis. As high Lef1 expression is already present in pre-leukemic samples (Figure 3A), it is likely that deregulated Wnt signaling predisposes thymocytes to induction of activating somatic mutations in Notch1, which subsequently accelerate lymphoma development.

### Wnt-Reporter Activity Is Minimally Present in Normal Tcf1^−/−^ Thymocytes and Enhanced in Tcf1^−/−^ Lymphoma Cells

**Figure 4:**
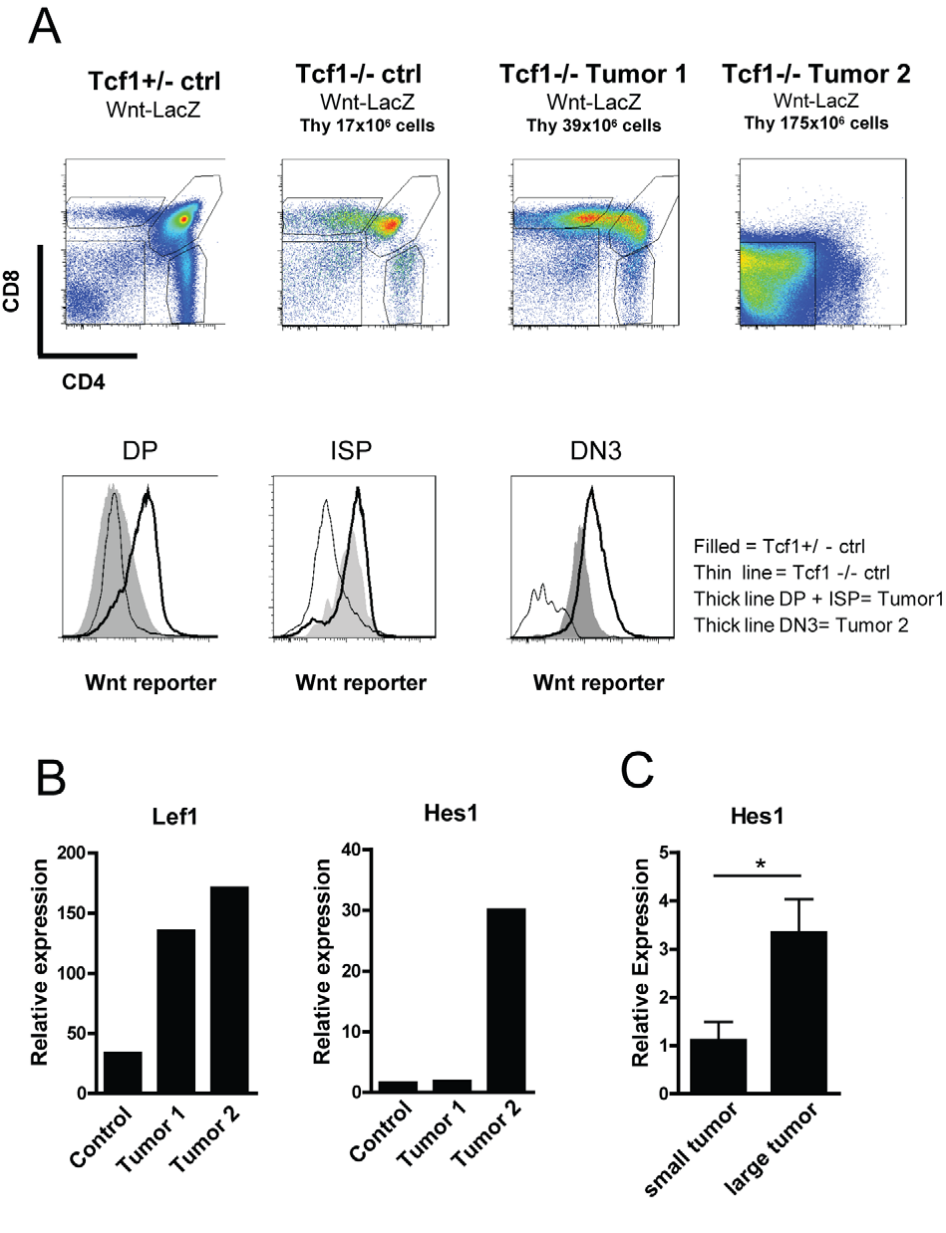
Blocked Tcf1^−/−^ thymocytes show high Wnt-signaling in lymphoma development. (A) Tcf1^−/−^ mice were crossed to Conductin(Axin2)-LacZ reporter mice and sacrificed at different ages. Dot plots of four representative mice are shown: one control Tcf1^+/−^ LacZ (age 20 wk), one control Tcf1^−/−^ LacZ (age 9 wk), and two Tcf1^−/−^ Axin2-LacZ mice (age 20 wk, Tumor 1+ Tumor 2). Organs were collected and thymocytes were stained for CD4, CD8, CD3, CD44, and CD25 together with FDG to demonstrate Wnt reporter activity in the different thymocyte subsets. The corresponding thymus sizes for the four mice shown are 110×10^6^, 17×10^6^, 39×10^6^, and 175×10^6^ cells, respectively. Expression of Wnt reporter activity is shown per thymocyte subset, DP, ISP, and DN3 for control cells (filled), Tcf1^−/−^ cells (thin line), and Tcf1^−/−^ tumor cells (thick line). (B) RNA was isolated of total thymus and the expression level of Lef1 and Hes1 relative to Abl is shown for the control thymus and the two tumor samples as shown in (A). (C) The mean expression levels of Hes1 relative to Abl are shown for small tumors (*n* = 5) and large tumors (*n* = 25).

To confirm the paradoxical finding that mice lacking Tcf1 suffer from thymic lymphomas due to deregulated high Wnt signaling rather than low, we crossed Tcf1^−/−^ mice with a well-established Wnt-reporter mouse strain, namely the Axin2-LacZ mice. Wnt-activity in these mice can be measured by the expression of β-galactosidase driven by the Axin2 promoter. In Figure 4A, the CD4/CD8 dot plots are shown of thymocytes of four different representative mice. The histograms show the Wnt-activity in DP, ISP, and DN3 cells for Tcf1^+/−^ thymocytes (filled), Tcf1^−/−^ thymocytes (thin line), and tumor Tcf1^−/−^ thymocytes (thick line). The thymocyte subsets of a Tcf1^−/−^ control mouse without a tumor show severely reduced Wnt-activity in ISP and DN3 thymocyte subsets compared to the Tcf1^+/−^ control mouse (Mean Fluorescence Intensity [MFI] of 385 and 104 compared to 874 and 635 in control ISP and DN3, respectively), indicating a strongly diminished nuclear response to Wnt signals due to the Tcf1 deficiency. Interestingly, residual Wnt-activity can be measured in Tcf1^−/−^ thymocytes, which suggests that Lef1 is mediating low levels of Wnt-activity in Tcf1^−/−^ mice as a likely compensatory mechanism (as also shown by Figure 3A). Tcf1^−/−^ mice developing lymphomas show enhanced Wnt activity in the developmental stages in which the tumor cells are blocked (MFI of 1,425 and 1,225 for Wnt-reporter signal in DP and ISP for tumor 1 and 2,123, 2,374, and 1,203 in DP, ISP, and DN3 for tumor 2). The thymi of the Tcf1^+/−^ control mouse and the two Tcf1^−/−^ tumor mice displaying high Wnt activity were further examined for the RNA expression levels of Lef1 and Hes1. The expression level of Lef1 was increased in both Tcf1^−/−^ induced lymphomas, indicating that these high levels of Lef1 underlie the highly active Wnt signals in these tumors (Figure 4B). Interestingly, only in tumor 2 (>175×10^6^ cells) were high levels of the Notch target gene Hes1 observed, indicating that Notch signaling accelerates or maintains tumor development once it is initiated by deregulated Wnt-signaling. Indeed, when we compared the thymus size to the expression level of Hes1, we found that only in large Tcf1^−/−^ tumors (>25×10^6^ cells) is the expression level of Hes1 increased (Figure 4C). These data suggest that a first oncogenic hit is the deregulation in Wnt-signaling due to high levels of Lef1 and that deregulation of the Notch-pathway is a secondary acquired mutation. To check for mutations in Notch1, we sequenced both the heterodimerization domain (HD), exon 26 and 27, and the PEST domain, encoded by exon 34. This analysis showed that in Tcf1^−/−^ thymi (without tumor) and Tcf1^+/−^ samples, no mutations were found in the three exons (*n* = 8, unpublished data). Analysis of the panel of 40 Tcf1^−/−^ thymic lymphomas demonstrated mutations in exon 34 in all but one thymic lymphoma sample (unpublished data), which is known to promote Notch1 signaling by increasing the half-life of intracellular Notch, hence promoting tumor survival and growth. Together, these results suggest that Tcf1 deficiency leads to a pre-leukemic stage that favors additional mutations, most notably in Notch1.

### Tcf1 Is the Major TCF/LEF Factor in the Thymus and Its Short Isoforms Function as Repressors of Lef1 Expression

**Figure 5:**
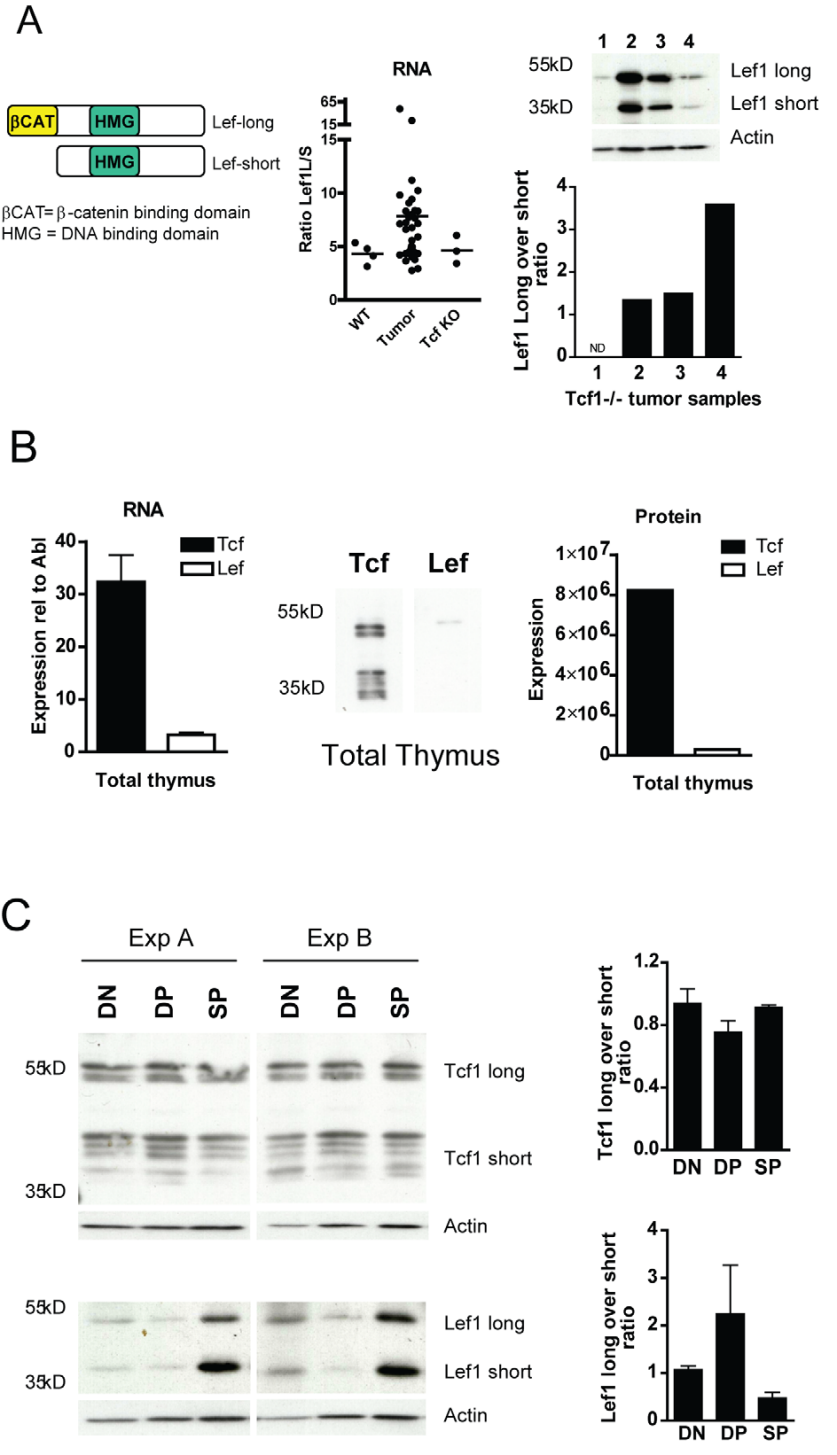
Lack of Tcf1 deregulates the balance between long and short isoforms of Lef1 in Tcf1-deficient lymphomas. (A) A schematic representation of the long and short isoform of Lef1, depicting the β-catenin binding domain and the HMG-box (DNA binding domain). The ratio of Lef1 long isoform versus Lef1 short isoform was determined using RQ-PCR for 40 lymphomas, four control thymi, and three Tcf1^−/−^ thymi without tumor. The long versus short ratio for Lef1 at the protein level is depicted for four different Tcf1^−/−^ thymic tumors (tumor size: 19, 224, 118, 30×10^6^ cells/ml, respectively). (B) Left panel, total Tcf and Lef RNA levels relative to Abl were determined using the data obtained from the Affymetrix data shown in Figure 3A (Tcf1^+/−^ total thymus, *n* = 5). In the middle panel, a Western blot analysis and quantification (right panel) for nuclear protein extracts of total thymocytes (Tcf1^+/+^ mouse) is shown. (C) Representative Western blots of two independent experiments (Exp A and Exp B) of sorted thymocyte subsets from two control Tcf1^+/+^ mice. Cell populations were sorted into the subsets DN, DP, and SP using lineage markers; CD3, CD4, CD8, CD44, and CD25 and total protein extracts were generated. Antibodies recognizing all isoforms of Tcf1 and Lef1 were used, and β-actin was used a loading control for the Western blots. Quantification of the Tcf and Lef signal of all Western blots was performed by ImageQuant J.

To gain further insight into the mechanism underlying lymphomagenesis, the balance between the long and short isoforms of Lef1 and Tcf1 was investigated. It is known that the balance between the long and short isoforms of these factors is crucial in regulating Wnt signaling, as only the long isoform can bind β-catenin and hence mediate Wnt signaling, whilst the short isoform is considered the natural antagonist of Wnt signaling (a simplified version of the short versus the long form of Lef is shown in Figure 5A). Analysis of the Tcf1^−/−^ tumor samples (RNA and protein) revealed that the normal ratio of long over short isoforms for Lef1 was altered in favor of the long isoform of Lef1, which mediates transcription of Wnt-β-catenin target genes (Figure 5A). The RNA levels of Tcf1 and Lef1 in normal thymus indicated a 10 times higher expression of Tcf1 than Lef1 in the thymus (Figure 5B). This is further illustrated by an example of the protein levels of Tcf1 and Lef1 in a nuclear extract protein sample of total thymocyes (Figure 5B, right panel). Reciprocal regulation of Tcf1 and Lef1 at the protein level was further examined in the major sorted thymic subsets (DN, DP, and SP). Of interest, the ratio between long Wnt responsive isoforms and short repressor isoforms is different for Tcf1 versus Lef1. For Tcf1, there is a clear expression of the inhibitory short isoforms in all thymocyte subsets, whilst for Lef1 in all stages the long β-catenin binding form is more abundant, except for the single-positive stage (Figure 5C). This suggests that major repressors of Wnt signaling in the thymus are formed by the Tcf1 short isoforms. Therefore, in the absence of Tcf1, a repression of Lef1 expression in the thymus is diminished. This allows for high levels of Lef1, which naturally occurs in a ratio of more Wnt responsive than inhibitory isoforms, hence strengthening the Wnt responsiveness of the (pre)leukemic cells. Thus, a major function of Tcf1 appears to control Lef1 expression via its short isoforms.

### Deregulated Wnt Signaling Frequently Cooperates with Deregulated Notch Signaling in Tcf1^−/−^ Induced Lymphomas

**Figure 6:**
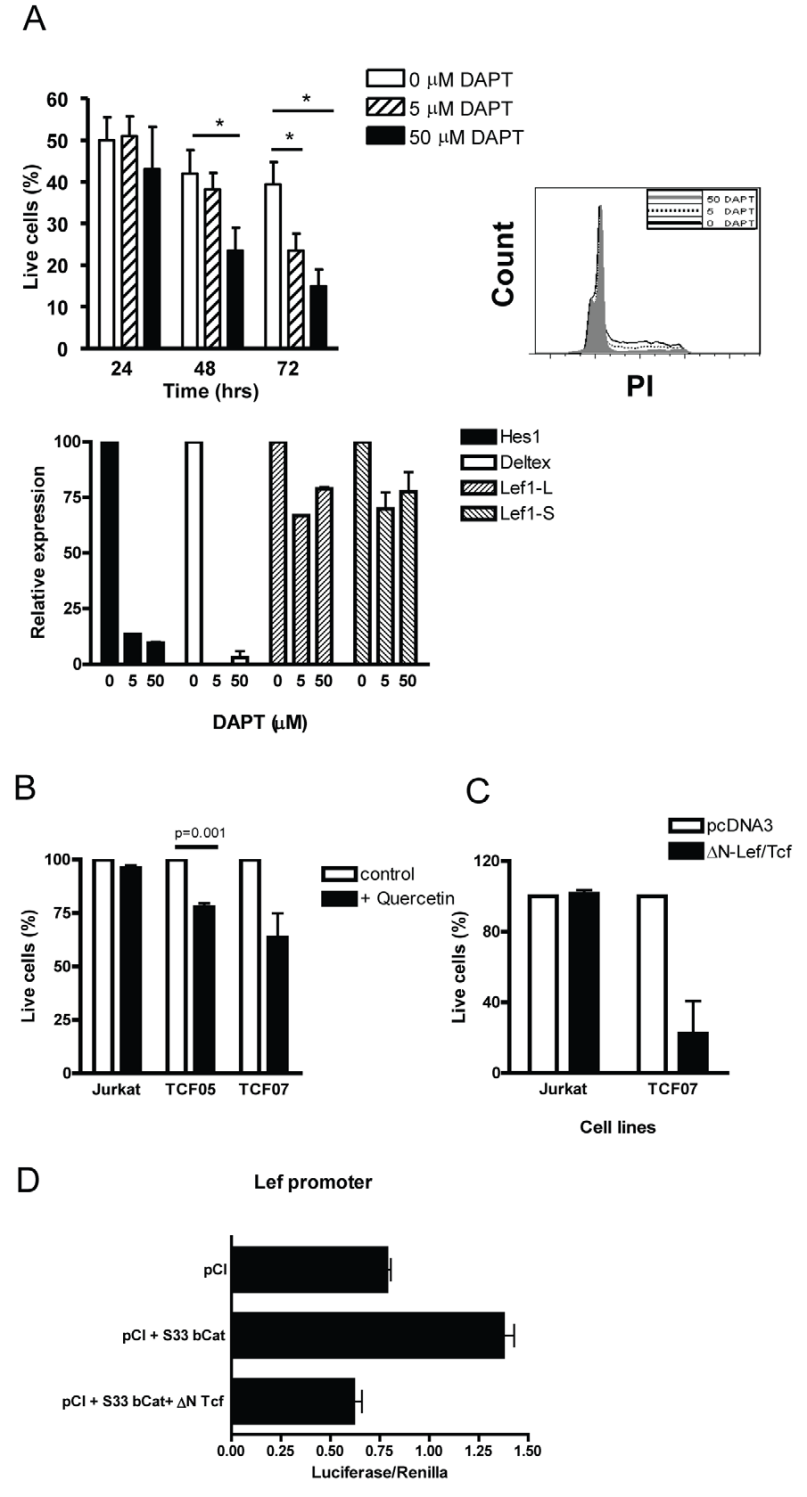
Tcf1-deficient lymphoma cells depend on Wnt and Notch signaling for their survival. Several Tcf1^−/−^ cell lines were established from Tcf1^−/−^ thymic lymphomas, and all cell lines show Notch1 mutations and a high ratio of Lef1 long over short isoform. (A) The TCF05 cell line (phenotyped as an ISP tumor) was cultured in the absence and presence of the indicated concentrations of γ-secretase inhibitor (DAPT). Percentage of live cells was determined after 24, 48, and 72 h using flow cytometry (AnnexinV/7AAD staining). After 24 and 48 h cell cycle analysis was determined by propidium iodide staining, and the relative expression of Hes1, Deltex, Lef1-L, and Lef1-S was determined. For the cell cycle analysis, one representative example is shown and the mean expression levels relative to Abl are shown of two independent experiments. (B) Three cell lines (Jurkat, TCF05, and TCF07) were cultured for 7 h in the presence or absence of Quercetin (25 µM), and the percentage of live cells was determined by flow cytometry (7AAD/AnnexinV staining). The mean percentage (± SEM) of live cells (7AAD−/AnnV−) is shown for the three cell lines of three independent experiments. (C) The Tcf1^−/−^ cell line TCF07 was established from a thymic lymphoma characterized as a DN3 tumor with a mutation in exon 34 of Notch1. This cell line and Jurkat cells were transfected with a GFP construct in combination with a control construct (pcDNA3) or a dominant negative Lef1/Tcf1 (pcDNA ΔNTCF) construct (used ratio GFP∶construct, 1∶10). The percentage of viable cells was determined within the transfected (GFP+) cells after 6 h. (D) HEK 293 T cells were transfected with a LEF-1 luciferase reporter plasmid containing Tcf/Lef-responsive elements. The cells were cotransfected with S33-Bcat or pCI (3 µg) or Delta N Tcf1 together with S33 B cat. To control for transfection efficiency, all transfections included the pRLTK-renilla reporter (0.15 µg). Luciferase activities are shown as mean of three independent experiments normalized to renilla activity.

The data described above suggest that both deregulated Wnt as well as Notch signaling are required for development of the Tcf1-deficient lymphomas. To investigate the Wnt and Notch dependency, we performed a number of experiments with pharmacological drugs and genetic tools. Using the γ-secretase inhibitor DAPT, a potent Notch1 inhibitor, clear loss of survival and proliferation was observed in Tcf1^−/−^ tumor cells (Figure 6A, left figure). Moreover, the high Lef1 levels in the tumor cells appeared not to be a result of the deregulated Notch signaling, as inhibition of the Tcf1^−/−^ tumor cells by DAPT only mildly affects Lef1 levels, whilst both Notch target genes *Hes1* and *Deltex* were completely down-regulated (Figure 6A, right figure). Ongoing activation of the Wnt signaling pathway was shown to be crucial for the survival of the Tcf1^−/−^ tumor cells in two different sets of experiments. First of all, incubation of the generated Tcf1^−/−^ cell lines with the Wnt inhibitor Quercetin, which blocks the interaction between β-catenin and Tcf/Lef factors, induces rapid cell death (7 h), whilst the non-Wnt-dependent Jurkat cell line was minimally affected (Figure 6B). In addition, transfection of a dominant negative form of Tcf1/Lef1 in Tcf1^−/−^ lymphoma cells also induced rapid cell death (>80% cell death after 6 h; Figure 6C). Thus, both ongoing Wnt and Notch signaling are required for the survival of Tcf1-deficient lymphomas.

To further investigate the Wnt and Lef1 dependency, we performed reporter gene analysis using the natural Lef1 promoter, which contains four consensus Tcf/Lef binding sites (Figure 6D). The luciferase experiments with the Lef1 promoter show that this promoter is Wnt responsive and demonstrate that Lef1 expression can be up-regulated by β-catenin-Lef1 complexes, providing a positive feedback loop. Such a positive feedback loop has been suggested before [25]. Consistent with the Wnt responsiveness, transfection of a ΔN Tcf1 construct, which acts as a dominant negative competitor, was capable of abolishing the β-catenin-induced activation of Lef1-dependent transcription. Thus, the Lef1 promoter is Wnt responsive and negatively regulated by short Tcf1 isoforms that lack the β-catenin interaction domain. Taken together, these data indicate that the highly deregulated Wnt signaling in the tumor cells is driven by Lef1; that it is frequently associated with increased Notch signaling, which acts as a collaborative oncogenic event; and that it is continuously required for survival of these lymphomas.

## Discussion

**Figure 7:**
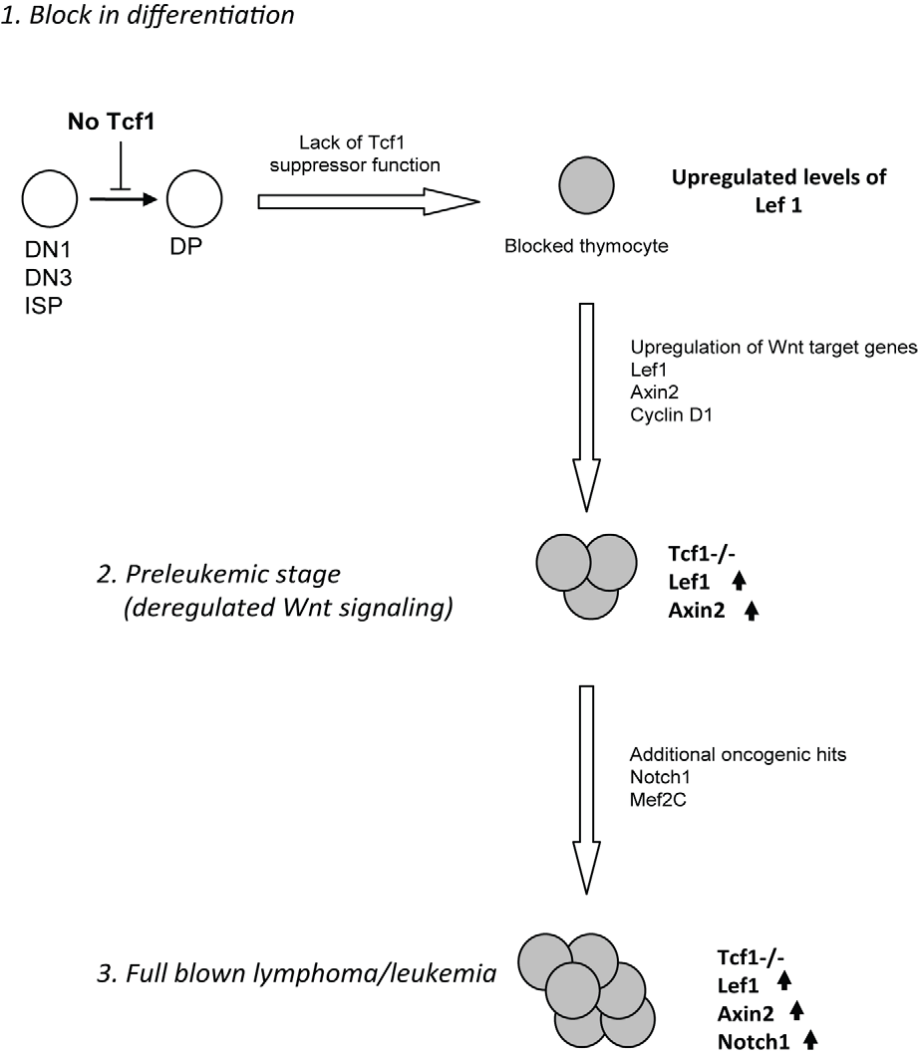
Mechanism of lymphomagenesis caused by Tcf1 deficiency. In the absence of Tcf1, thymocyte development is blocked at several stages (DN1, DN3, and ISP). Arrested thymocytes lack all isoforms of Tcf1, including the repressive isoforms. Loss of these repressive isoforms results in an up-regulation of Lef1, of which the long isoforms are most abundant in the thymus. Subsequently, Lef1 is capable of interacting with β-catenin as a compensatory mechanism, inducing deregulated Wnt signaling as measured by the high expression of *Axin2* in the vast majority of the lymphomas, forming a pre-leukemic stage. After additional mutations are acquired, of which activating mutation in *Notch1* frequently occur, and also other oncogenes such as *Mef2C* and *Lmo2* are likely candidates to be affected, full-blown lymphoma/leukemia develops.

During normal hematopoiesis, Tcf1 is required to induce and maintain proliferation of developing T cells in the thymus [19] and inhibit apoptotic signals at the DP stage [23]. Absence of Tcf1 not only results in a severely reduced thymic cellularity but also blocks differentiation of the thymocytes [11],[19],[21]. It is remarkable that Tcf1^−/−^ cells blocked in differentiation develop into lymphoma cells, as we here report. We propose that Tcf1^−/−^ blocked thymocytes give rise to lymphoma cells due to deregulated Wnt signaling, which is driven by expression of deregulated expression of Lef1. This model is based on several key observations (Figure 7). First of all, in essentially all Tcf1^−/−^ tumors, a high expression of *Lef1* (selectively of the long form of Lef1, which is able to bind β-catenin) was found. This up-regulation of Lef1 likely acts as a compensatory mechanism for the lack of Tcf1 and is probably caused by lack of repression by Tcf1. We show much higher Tcf1 than Lef1 expression in the normal thymus, including the naturally occurring dominant negative isoforms of Tcf1. This suggests a direct repressor function of Tcf1 for Lef1 expression given the normally much higher Tcf1 than Lef1 expression in thymus. Especially since Lef1 has more long-beta catenin responsive isoforms (except in the SP stage), while Tcf1 has slightly more expression of the short form. Hence in the complete absence of Tcf1, Lef1 will take over as a Wnt-responsive transcription factor in the thymus. Thus, the lymphoma development is initiated by developmental arrest due to lack of Tcf1; thereby, the suppression of Lef1 expression by short Tcf1 isoforms is lifted. This leads to higher Lef1 expression and a propensity to higher Wnt responsiveness, restoring proliferation and increasing Wnt target gene expression and concomitantly to the possibility of induction of somatic mutations, such as those found in Notch1. Lef1 expression can be further enhanced by Notch, as shown in lymphomas that lack the E2A transcription factor [27]. Moreover, Lef1 can also positively regulate its own expression through Wnt dependent ([26], plus data in Figure 6D) and independent mechanisms [26],[28]. Finally, up-regulation of other oncogenes will lead to frank lymphoma/leukemia development. In this respect, up-regulation of T-ALL oncogenes such as *Lmo2* and *Mef2C* in the Tcf1^−/−^ thymocytes may also contribute to the preleukemic nature of these cells. Importantly, the in vivo evidence for this model was provided by crossing Tcf mice with Axin2-LacZ-reporter mice. Tcf1^−/−^ mice without tumors have a reduced level of Wnt-activity in all thymocyte subsets compared to Tcf1^+/−^ mice, whilst Tcf1^−/−^ thymic lymphoma cells show a very high level of Wnt-activity in blocked thymocytes.

It is of interest to compare the Tcf1^−/−^ lymphomas with two other murine lymphoma models, namely those induced by activated β-catenin and by lack of E2A. The development of Tcf1^−/−^ lymphomas contrasts with thymic lymphomas induced by overexpression of β-catenin in that the latter show no Notch1 mutations [29] but may be dependent on p53 absence [30]. In the E2A-deficient lymphomas, Lef1 is a Notch target gene only in the context of the lymphoma cells, but not in normal progenitors, and Lef1 is essential for lymphomagenesis. Thus, in the absence of normal regulatory mechanisms in thymic lymphoma cells provided by E2A or Tcf1, Lef1 can act as an important oncogene. Interestingly, the Tcf1^−/−^ thymic lymphomas easily gain additional mutations in the *Notch1* gene (in contrast to the β-catenin-dependent lymphomas), which leads to further development of these lymphomas. Once Notch1 expression is established, it may serve as an accelerator of the Lef1-mediated deregulated Wnt signaling, ensuring increased survival and expansion of the lymphoma cells [27].

In the human equivalent of these T-cell lymphomas, T-cell acute lymphoblastic leukemias (T-ALL), several genetic abnormalities have been described including Notch1 mutations in a large proportion of all human T-ALL [31]. While it is difficult to unravel the stepwise process of leukemia development in humans, the activating mutations in Notch1 are not always the initiating events as shown by data from a leukemia that was observed in a gene therapy trail for X-linked SCID [32]. In this case, it was conclusively shown that insertional mutagenesis near the LMO2 proto-oncogene was the first genetic aberration followed by Notch1 mutations and further genetic aberrations [32]. It will be of high interest to see if loss of Tcf1 tumor suppressor function occurs in human T-ALL. Whether loss of function of Tcf1 as a tumor suppressor gene actually occurs in human T-ALL is currently under investigation. Human T-ALL with mutations in Lef1 have been described, although the mechanistic consequence of these mutations is currently unclear [33].

Two recent studies from the Bhandoola and Gounari laboratories, respectively, have conclusively demonstrated a key role for Tcf1 in establishing T-cell commitment [10],[16].

Deletion of β-catenin in the thymus has been reported both to affect T-cell development (using Lck-CRE) or to have no effect at all, when uisng *Mx*-Cre-mediated deletion of β-catenin [34] or β- and γ-catenin simultaneously [35],[36]. However, our recent work on Wnt dosage in various hematopoietic lineages including thymocytes suggests that the lack of phenotype using mx-Cre might be caused by the fact that Wnt signaling was not completely abolished in these models [37],[38]. Moreover, the Held Group also published that the Tcf1 phenotype can be complemented by transgenic expression of a long Tcf1 isoform but not by a short (non/Wnt responsive) isoform [23]. Therefore, a major role for Tcf1 in the thymus is to integrate Wnt responsive signals and thereby allow T-cell development to occur normally. Nevertheless, our current work also indicates an important non/Wnt-dependent role of TCF, as a negative regulator of the Wnt pathway. The recent work of Bhandoola is also interpreted as a non/Wnt-dependent effect. Therefore, it is an intriguing possibility that both Wnt-dependent effects of Tcf1 (e.g., Wnt-driven proliferation of DN thymocytes) and Wnt-independent effects (induction of T-cell specification) collaborate in the early stages of T-cell development.

In summary, we here report that Tcf1 has a dual function during T-cell development: first, it is needed as a transcriptional activator of Wnt-induced proliferation, but unexpectedly it also acts as a transcriptional repressor and tumor suppressor gene to prevent the development of thymic lymphomas and it may also function in a Wnt-independent way in these early stages of T-cell development, as suggested by recent work [16] perhaps by repressing genes of alternative (non-T) lineages. We conclude that Tcf-1 acts as a molecular switch between proliferative and repressive signals during T-cell development in the thymus.

## Materials and Methods

### Mice

C57Bl/6 Tcf1^−/−^^ΔVII/ΔVII^ were originally described by Verbeek [19], C57Bl/6-CD45.1 (Ly5.1) and C57Bl/6-Rag1^−/−^ mice were obtained from the Jackson Laboratory, and Conductin(Axin2)-LacZ mice were kindly provided by B. Jerchow and W. Birchmeier (Max Delbrück Center for Molecular Medicine, Berlin, Germany) [39]. All mice were kept in the specified pathogen-free (SPF) breeding section, and this study was approved by the institutional Animal Ethical Committee of the Erasmus MC, Rotterdam and the Leiden University Medical Center, Leiden.

### Immunohistochemistry

Paraffin sections of organs of Tcf1^−/−^ mice were stained with H&E or with antibody against CD3 (A045229; DAKO, Glostrup, Denmark) and biotinylated goat anti-rabbit IgG (BA-1000; Vector Labs, Burlingame, CA, USA) as the secondary antibody. Visualization was enforced with ABC staining kit (PK6100, Vector Labs) with 3,3′-diaminobenzidine tetrahydrochloride (DAB, D5637, Sigma-Aldrich, St Louis, MO, USA) as substrate. Mayer's hematoxilin was utilized as nuclear counterstaining.

### Flowcytometry

The following antibodies were obtained from BD Biosciences (San Diego, CA): anti-CD3-APC (145-2C11), anti-CD4-PeCy7 (RM4-5), anti-CD8-PerCP(53-6.7), anti-CD25-PE (PC61), anti-CD44-PE (IM7), anti-CD24-FITC (M1/69), anti-cKit-PeCy7 (2B8). Lineage markers Mac1 (M1/70), Gr1 ((RB6-8C5), B220 (Ra3-6B2), and Ter119 and Nk1.1 (PK136) were all biotinylated and streptavidin APC-Cy7. Cells were stained in Fluorescence-activated cell sorter (FACS) buffer (PBS, 2% bovine serum albumin, 0.1% sodium azide) for 30 min at 4°C. Intracellular β-galactosidase activity was measured by staining cells with 2 mM fluorescein di-β-D-galactopyranoside (FDG) substrate (Molecular Probes). FDG was loaded into the cells by hypotonic shock at 37°C for 1 min, prior to cell surface antibody staining. The β-galactosidase reaction was stopped with 1 mM phenylethyl β-D-thiogalactopyranoside (PETG, from Molecular Probes). Cells were washed and immediately analyzed on a Canto I (BD Biosciences). Data were analyzed using FlowJo software (Tree Star, Ashland, OR, USA).

### In Vivo Tumor Induction

Lymphoma or thymocyte suspensions of previously characterized mice were prepared aseptically. Cells (5×10^5^) were injected in the tail vein of sublethally irradiated (4 Gy) Rag1^−/−^ mice. The mice were bled every 4 wk until the end of the experiment. The presence of lymphoma cells was investigated by flow cytometry.

### Southern Blot Analysis

DNA (10 µg) was digested with *Eco*RI overnight at 37°C, separated on a 0.7% agarose gel, and blotted onto a positively charged nylon membrane (Hybond, Amersham). Southern blots were probed with a ^32^P-labeled 1.2 kb *Eco*RI-*Cla*I genomic fragment recognizing the Jβ2 region of the TCRβ gene.

### Western Blot Analysis

Total protein lysates and nuclear extracts were generated from total thymocytes and sorted populations (DN, DP, and SP). Total protein lysates were generated by immediately lysing the cells in boiling sample buffer (150 mM Tris-HCl pH 6.8, 300 mM DTT, 30% glycerol, 6% SDS, 0.1% bromophenol blue). Nuclear extracts were generated by resuspending cells in buffer A (10 mM Hepes, 1.5 mM MgCl2, 10 mM KCl, 1 mM EDTA, 0.5 mM DTT and freshly added protein inhibitor cocktail [PIC]) for 15 min on ice. Subsequently NP40 (final concentration 0.6%) was added, thoroughly mixed, and the cytoplasmic extract was removed by centrifugation. Remaining nuclei were lysed by incubating with buffer C (20 mM Hepes, 10% glycerol, 1.5 mM MgCl2, 0.42 M NaCl, 0.2 mM EDTA, and freshly added DTT and PIC) for 30 min at 4°C. Nuclear extracts were ready after centrifugation. Protein concentration was measured using BCA Protein Assay kit (Pierce, Rockford, MD, USA). Lysates containing 1 µg of protein were separated by electrophoresis on a 10% SDS-polyacrylamide gel and transferred onto PVDF membranes. Nonspecific binding was blocked by incubation in blocking buffer (2.5% BSA in TBS-Tween) followed by incubation with the primary antibodies and the appropriate secondary antibodies conjugated to horseradish peroxidase. All isoforms of Tcf1 were detected by anti-Tcf1 antibody (clone C46C7, rabbit mAb, Cell Signaling, Boston, USA), and all Lef1 isoforms were detected by anti-Lef1 antibody (clone C18A7, rabbit mAb, Cell Signaling). Equal loading was confirmed by reprobing the blots with an anti-actin antibody.

### Microarray Analysis

Thymocytes were homogenized for RNA isolation using Qiagen RNeasy minicolumns. The quantity and quality of total RNA was determined using spectrophotometry (Nanodrop) and an Agilent Bioanalyzer. One µg of RNA was used to generate cRNA using Affymetrix One cycle cDNA synthesis kit (Affymetrix, Santa Clara, CA, USA), after which the samples were biotinylated using an Affymetrix IVT labeling kit (Affymetrix). The samples were hybridized overnight at 42°C to GeneChip mouse genome 430 2.0 Arrays (Affymetrix). Washing and staining steps were performed on a Fluidics station 450, and the Genechips were scanned using a GeneChip scanner 3000 (Affymetrix) at the Department of Immunology, Erasmus Medical Center. Raw data were normalized and summarized using Robust Multichip Average (RMA) method [40]. Array analysis was performed using R-2.14 (http://cran.r-project.org/) and Bioconductor 2.9 software (http://www.bioconductor.org/) using the bpca [41] and gplots [42] packages. From the dataset, genes were selected for display in heatmaps, in which the rows of the expression matrix were ordered by hierarchical clustering of Eucledian distances between the samples, with the expression intensities being scaled per probeset. Principal component analysis was performed on a dataset of Tcf1^+/−^, Tcf1^−/−^, and Tcf^−/−^ tumor samples, using selection of Wnt and Notch response genes (*Emp1*, *Tcf7*, *Tnfrsf19*, *Hes1*, *Dtx1*, *Notch1*, *Axin2*, *Lef1*, *Cd44*, *Runx2*, *Fn1*, *Cdh1*, *Jun*, *Ccnd1*, *Krt8*, *Id2*, and *Jag1*). The first three principal components are displayed.

### Real-Time Quantitative-PCR Analysis (RQ-PCR)

Total RNA was extracted using Qiagen RNeasy minicolumns. One µg of total RNA was used as a template for cDNA synthesis, using Superscript II reverse transcriptase (Invitrogen, Carlsbad, CA, USA), Oligo dT, and random hexamer primers. The RQ-PCR reaction was performed using TaqMan Universal mastermix (Applied biosystems, Foster City, CA, USA) and was run on a PRISM 7700 sequence detection system containing a 96-well thermal cycler (Applied Biosystems). The following primers were used in combination with FAM-labeled probes from the universal probe library (Roche): Deltex1 forward primer: 5′-GAAGAACTTGAATGGCACTGG-3′; reverse primer: 5′-GTTTGGGTGCTCGTGTCAG-3′; Lef1 short forward primer: 5′-GCGACACTTCCATGTCCAG-3′; reverse primer: 5′-TCCTGTTTGACCTGAGGTGTTA-3′; Lef1 long forward primer: 5′-TGGTTAACGAGTCCGAAATCA-3′; reverser primer: 5′-AGAGGACGGGGCTTGTCT-3′; Axin2 forward primer: 5′-GCAGGAGCCTCACCCTTC-3′; reverse primer: 5′-TGCCAGTTTCTTTGGCTCTT-3′; Hes1 forward primer: 5′-AAACACTGATTTTGGAGCACT-3′; and reverse primer: 5′-TGCTTCACAGTCATTTCCAGA-3′. RQ-PCR results were normalized to *Abl* expression in the same sample: forward primer: 5′-TGGAGATAACACTCTAAGCATAACTAAAGGT-3′; reverse primer: 5′-GATGTAGTTGCTTGGGACCCA-3′; and probe: 5′-FAM-CCATTTTTGGTTTGGGCTTCACACCATT-TAMRA-3′.

### Notch1 Mutation Analysis

cDNA of total thymus was used for the amplification of exons encoding the Notch1 heterodimerization and PEST domains. Primers used for the identification of activating Notch1 mutations are described elsewhere [43].

### Tcf1^−/−^ Cell Line Experiments

Several Tcf1^−/−^ cell lines were established from Tcf1^−/−^ thymic lymphomas, and all cell lines show Notch1 mutations and a high ratio of Lef1 long over short isoform. Transfection experiments were performed by transfecting the cell line with eGFP together with either a control construct (pcDNA3) or a dominant negative Lef1/Tcf1 construct (transfection ratio GFP∶construct, 1∶10) using AMAXA electroporation technology. Transfected cells were identified based on GFP positivity and phenotype, and cell viability was determined 6 h after transfection. Discrimination between viable and dead cells was performed by staining the cells with AnnexinV and 7AAD (BD Bioscience). Tcf1^−/−^ cell line cultures were performed in the presence and absence of the γ-secretase inhibitor DAPT (0, 5, and 50 µM) or Quercetin (50 µM). At the indicated time points, cell cycle analysis was performed using propidium iodide, live cells were determined by 7AAD/AnnexinV stain, and RNA was isolated for gene expression levels.

### Luciferase Reporter Gene Assays

293 T cells were cultured in Iscove's Modified Dulbecco's Medium supplemented with 10% fetal bovine serum, L-glutamin, and penicillin/streptomycin and transfected using the Fugene method according to the manufacturer's procedures (Roche). The cultures were transfected with 1.5 µg LEF-1 4000 luciferase reporter plasmid (containing four Tcf/Lef-responsive elements) or 1.5 µg of the LEF-1 600 luciferase reporter plasmid (all Tcf/Lef-responsive elements deleted) (kindly provided by Dr. J. Skokowa, Hannover Medical School [44]). The cells were cotransfected with S33-βcatenin and/or pCI and/or ΔN-Tcf (3 µg). To control for transfection efficiency, all transfections included the pRLTK-renilla reporter (0.15 µg). Transfected cells were cultured for 24 h and then lysed and assayed for reporter activity. Luciferase and Renilla activity was measured using a dual-luciferase reporter assay system from Promega (Madison, USA). All luciferase activities were normalized to Renilla activities.

### Statistical Analysis

Statistical analysis was performed using the Mann–Whitney U test (Prism GraphPad Software, San Diego, CA, USA). *p*<0.05 was considered statistically significant.

## Abbreviations

DN: double negative
DP: double positive
ETP: early thymic progenitor
HMG: high mobility group
ICAT: inhibitor of the interaction between beta-catenin and Tcf/Lef factors
ISP: immatures single positive
Lef: Lymphocyte enhancer binding factor
MFI: mean fluorescence Intensity
Tcf: T cell factor
